# Association between number of dissected lymph nodes and survival in stage IA non-small cell lung cancer: a propensity score matching analysis

**DOI:** 10.1186/s12957-020-02090-5

**Published:** 2020-12-07

**Authors:** Lei-Lei Wu, Jia-Jian Lai, Xuan Liu, Yang-Yu Huang, Peng Lin, Hao Long, Lan-Jun Zhang, Guo-Wei Ma

**Affiliations:** 1 Sun Yat-sen University Cancer Center, State Key Laboratory of Oncology in South China, Collaborative Innovation Center for Cancer Medicine, Guangzhou, 510060 P. R. China; 2 Sun Yat-sen University, Guangzhou, 510060 P. R. China; 3 The Department of Thoracic Surgery, Sun Yat-sen University Cancer Center, 651 Dongfengdong Road, Guangzhou, 510060 P. R. China

**Keywords:** Non-small cell lung cancer, Lymph nodes, Prognosis, Surgery, Small tumor size

## Abstract

**Background:**

For patients with stage IA non-small cell lung cancer (NSCLC) with tumor size ≤ 2 cm, the prognostic significance of the number of removed lymph nodes (NLNs) through different surgical methods remains unclear. To determine the association of NLNs with cancer-specific survival (CSS) and overall survival (OS) in patients with stage IA NSCLC with tumor size ≤ 2 cm who underwent different lung surgeries.

**Methods:**

We retrospectively enrolled 7293 patients from the Surveillance, Epidemiology and End Results database. Median NLNs was used to classify the patients into two groups: group A with NLNs ≤ 5 and group B with NLNs > 5. Propensity score matching (PSM) was performed to decrease selection bias. Kaplan–Meier analysis and Cox regression analysis were performed to identify the association between NLNs and survival outcomes.

**Results:**

Group B had better survival than group A in the unmatched cohort and matched cohort (all *P* < 0.05). Multivariable analyses revealed that the NLNs significantly affected CSS and OS of eligible cases in the unmatched cohort and matched cohort. Additionally, we found that the NLNs was a protective prognostic predictor of OS for patients who underwent wedge resection, segmental resection, or lobectomy.

**Conclusion:**

The NLNs was a protective prognostic factor in NSCLC patients with tumor size ≤ 2 cm. We demonstrated that patients with > 5 NLNs in the cohort of wedge resection, segmental resection, or lobectomy exhibited a significantly better OS.

## Introduction

Lung cancer is one of the most aggressive malignancies worldwide. In 2019, lung cancer accounted for 13% of all estimated new cancer cases and one-quarter of all estimated cancer deaths in adults [1]. Patients with stage IA (according to 8^th^ American Joint Committee on Cancer [AJCC] Staging Manual) non-small-cell lung cancer (NSCLC) may undergo different surgical treatments, such as wedge resection, segmental resection, lobectomy, or pneumonectomy; however, their 5-year overall survival rate remains around 73–90% [2]. Lobectomy along with mediastinal lymph nodes (LNs) resection has been considered the standard surgical treatment for early-stage NSCLC for more than 2 decades [3]. LNs’ dissection is widely used to determine the accurate pathologic staging of NSCLC and to provide guidance regarding the prognosis and additional treatments. Multiple studies have indicated that LN resection conferred benefit in terms of the survival outcomes of patients with stage T1-4N0M0 NSCLC [4–8]. Furthermore, it seems that the dissection of more number of LNs may result in a clearer TNM classification and improve the survival outcomes of patients [9, 10].

Whereas with the popularization of computed tomography (CT)-based screening and application of some CT features, the predictive ability to distinguish benign and malignant lesions of small pulmonary nodules has significantly improved [11–13]. Patients with clinical stage I NSCLC commonly undergo lung cancer screening by CT, which helps detect the pulmonary nodules. Recently, the results of JCOG0804 demonstrated that for patients who were found with a lesion with a diameter ≤ 2 cm and a solid component ≤ 25%, the 5-year disease-free survival rate of those patients with sub-lobectomy was 99.7% [14]. Therefore, surgeons and patients might consider sub-lobar resection, including wedge resection, and segmental resection as a preferred treatment approach to protect more healthy tissues in the lung. However, with increased sub-lobar resection, the decision to include LN dissection remains controversial. In addition, few studies have investigated the relationship between LN resection and the survival outcomes of patients with clinical stage IA (8th AJCC) NSCLC with tumor size ≤ 2 cm who underwent different types of lung surgery (wedge resection, segmental resection, lobectomy, pneumonectomy) [7].

Therefore, we aimed to investigate whether resection of more LNs resulted in better survival outcomes among patients with clinical stage IA NSCLC with tumor size ≤ 2 cm and to determine the effects of the number of removed lymph nodes (NLNs) on conferring survival benefit to patients who underwent wedge resection, segmental resection, lobectomy, or pneumonectomy.

## Materials and methods

### Patients

This study was a retrospective study and approved by the Clinical Research Ethics Committee of Sun Yat-sen University Cancer Center (IRB number: B2019-116-01), and the need for informed consent of patients was waived. Between 2004 and 2015, 7293 patients with stage IA NSCLC and tumor size ≤ 2 cm from the Surveillance, Epidemiology, and End Results (SEER) database were retrospectively included in this study. The clinical TNM classifications were based on the 8th edition AJCC classification criteria. The inclusion criteria were as follows: (1) pathologically diagnosed as lung cancer (LC), (2) complete follow-up, (3) virtual survival status and clear survival time, (4) first malignancy and one primary, (5) diagnosis between 2004 and 2015, (6) primary non-small cell lung cancer, and (7) patients with clinical stage IA. The exclusion criteria were as follows: (1) Unknown 8th AJCC TNM stage, (2) diagnosed as small cell lung cancer, (3) stage other than IA, (4) tumor size more than 20 mm, (5) dead within 1 month, (6) did not receive lung resection, (7) follow-up of alive cases was within 60 months, (8) unknown information on lymph node resection, (9) had radiotherapy records, and (10) had chemotherapy records.

### Data collection

SEER is a population-based cancer registry that is made up of 18 geographically distinct tumor registries and covers 26% of the United States population. The data that supported the findings of this study are available in SEER Stat software (version 8.3.8), reference number: SEER 18 Regs Custom Data, Nov Sub (1975-2016 varying).

### Statistical analyses

The baseline characteristics between the two groups were evaluated using the chi-squared test, Fisher’s exact test, and Student’s *t* test. Both Kaplan–Meier analysis and the log-rank test were used to investigate the role of NLNs in cancer-specific survival (CSS) and overall survival (OS). Univariable and multivariable Cox regression analyses were performed to test the significant variables associated with CSS and OS. All statistical tests were two-sided, and probability values (*p* value) < 0.05 were considered statistically significant. Hazard ratio (HR) alongside 95% confidence intervals (CIs) was used to present the relative risk of the factors. Standard deviation (SD) was used to evaluate the stability of data in this cohort. The cutoff point of NLNs was determined using the median. Besides, to improve the test level and minimize the selection bias of the study, a 1:1 patient paired propensity score matching (PSM) analysis was performed. Age at diagnosis (age), sex, race/ethnicity, tumor size, TNM stage, grade, histological types, and surgical approaches were considered as covariates in the PSM model. With PSM, 2350 pairs were generated from 7293 eligible patients. All data in this study were analyzed using R (3.6.1) (https://www.r-project.org/).

## Results

### Patient characteristics

**Fig. 1:**
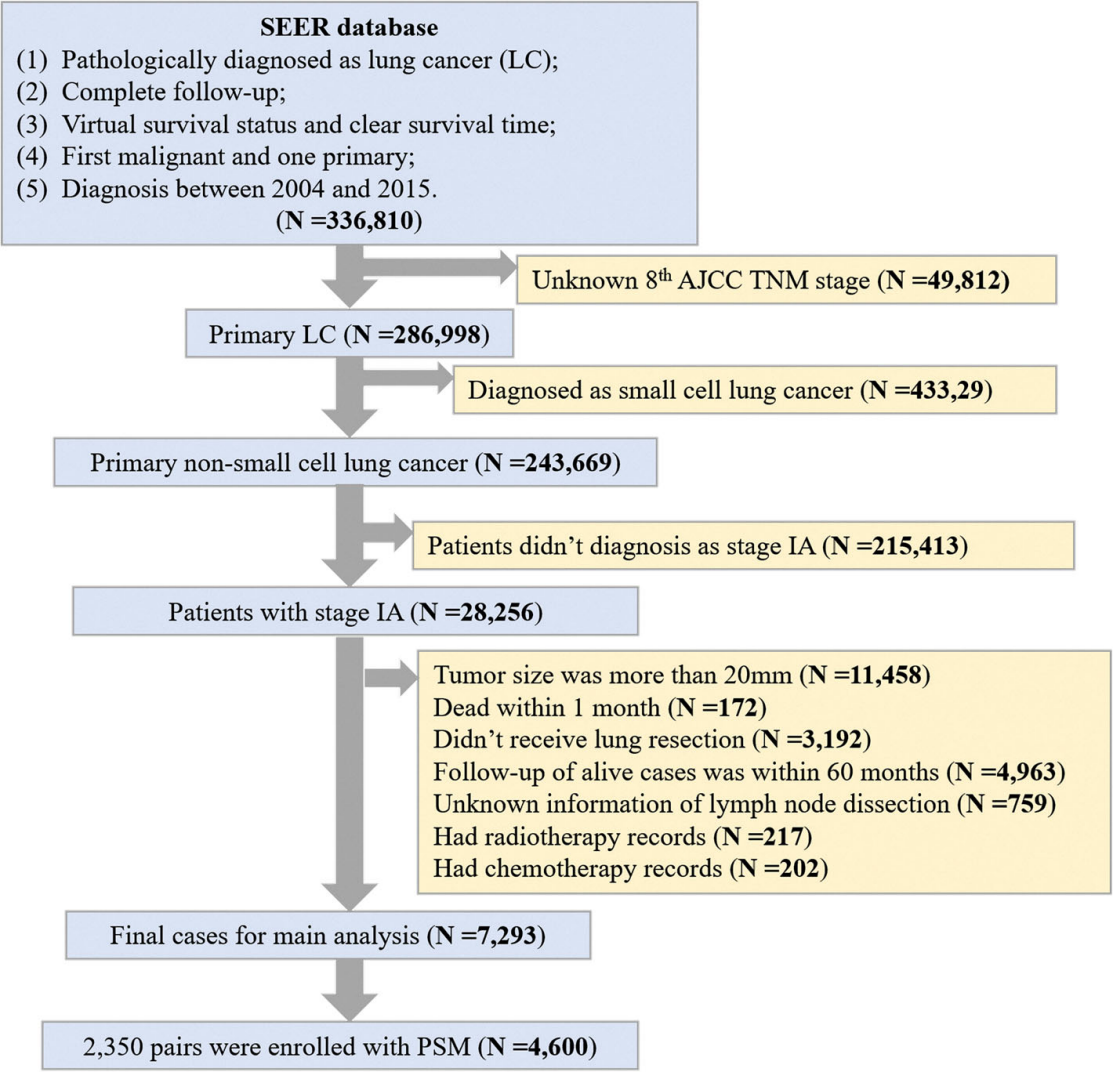
The flow diagram of this study

A total of 7293 eligible patients were enrolled in this study. The flow chart of this study is shown in Fig. 1. The median size of the tumor was 1.5 cm (range 0.1–2.0 cm). Similarly, the mean tumor size was 1.5 cm (SD 0.4 cm). Meanwhile, the median NLNs was 5 (range 0–78) and the mean NLNs was 6.72 (SD 6.88). The patients were divided into two groups according to the median NLNs. A total of 3850 patients had ≤ 5 LNs dissected, whereas 3443 patients had > 5 LNs dissected. Table 1 shows the baseline characteristics of patients stratified by NLNs (≤ 5 and > 5) before and after PSM. Before PSM, a significant difference between the two groups was seen in terms of age (*P* < 0.001), stage (*P* = 0.004), tumor location (*P* < 0.001), tumor size (*P* < 0.001), and surgical methods (*P* < 0.001). With PSM, 2350 pairs of patients were eligible for analysis and further stratified by the NLNs (≤ 5 and > 5). Because of PSM, both groups were well balanced as all the variables were not significantly different between groups.

**Table 1:** Baseline characteristics of patients with stage IA NSCLC ≤ 2 cm in size stratified by NLNs without and with PSM

	Without PSM			With PSM		
Groups	NLNs ≤ 5	NLNs > 5	*p* value	NLNs ≤ 5	NLNs > 5	*p* value
Total	3850	3443		2350	2350	
Sex (%)			0.437^*^			0.423^*^
Male	1588 (41.2%)	1452 (42.2%)		968 (41.2%)	940 (40.0%)	
Female	2262 (58.8%)	1991 (57.8%)		1382 (58.8%)	1410 (60.0%)	
Age (%)			< 0.001^*^			0.906^*^
≤ 65	1468 (38.1%)	1517 (44.1%)		990 (42.1%)	985 (41.9%)	
> 65	2382 (61.9%)	1926 (55.9%)		1360 (57.9%)	1365 (58.1%)	
TNM stage (%)			0.004^*^			1^*^
IA1	703 (18.3%)	541 (15.7%)		349 (14.9%)	350 (14.9)	
IA2	3147 (81.7%)	2902 (84.3%)		2001 (85.1%)	2000 (85.1%)	
Grade (%)			< 0.001^*^			0.707^*^
I	841 (21.8%)	774 (22.5%)		525 (22.3%)	530 (22.6%)	
II	1622 (42.1%)	1569 (45.6%)		1045 (44.5%)	999 (42.5%)	
III	992 (25.8%)	847 (24.6%)		578 (24.6%)	609 (25.9%)	
IV	55 (1.4%)	37 (1.1%)		32 (1.4%)	32 (1.4%)	
Unknown	340 (8.8%)	216 (6.3%)		170 (7.2%)	180 (7.7%)	
Tumor location (%)			< 0.001^**^			1^**^
Upper lobe	2258 (58.6%)	2289 (66.5%)		1388 (59.1%)	1395 (59.4%)	
Middle lobe`	311 (8.1%)	148 (4.3%)		148 (6.3%)	146 (6.2%)	
Lower lobe	1216 (31.6%)	965 (28.0%)		783 (33.3%)	777 (33.1%)	
Main bronchus	5 (0.1%)	4 (0.1%)		1 (0.0%)	1 (0.0%)	
Overlapping	16 (0.4%)	12 (0.3%)		10 (0.4%)	10 (0.4%)	
Lung NOS	44 (1.1%)	25 (0.7%)		20 (0.9%)	21 (0.9%)	
Tumor size (mean (SD) (mm))	14.72 (4.03)	15.05 (3.86)	< 0.001^***^	15.12 (3.88)	15.08 (3.81)	0.690^***^
Race (%)			0.059^**^			0.972^**^
American Indian	15 (0.4%)	14 (0.4%)		10 (0.4%)	12 (0.5%)	
Asian	194 (5.0%)	203 (5.9%)		141 (6.0%)	148 (6.3%)	
Black	324 (8.4%)	239 (6.9%)		197 (8.4%)	196 (8.3%)	
White	3308 (85.9%)	2983 (86.6%)		3 (0.1%)	4 (0.2%)	
Unknown	9 (0.2%)	4 (0.1%)		1999 (85.1%)	1990 (84.7%)	
Surgical methods (%)			< 0.001^**^			1^**^
Wedge resection	1417 (36.8%)	230 (6.7%)		230 (9.8%)	230 (9.8%)	
Segmental resection	284 (7.4%)	80 (2.3%)		79 (3.4%)	79 (3.4%)	
Lobectomy	2126 (55.2%)	3102 (90.1%)		2030 (86.4%)	2030 (86.4%)	
Pneumonectomy	17 (0.4%)	28 (0.8%)		11 (0.5%)	11 (0.5%)	
Unknown	6 (0.2%)	3 (0.1%)		0 (0.0%)	0 (0.0%)	
Histological types (%)			0.157^**^			0.866^**^
Adeno	2503 (65.0%)	2297 (66.7%)		1592 (67.7%)	1565 (66.6%)	
SCC	861 (22.4%)	745 (21.6%)		494 (21.0%)	510 (21.7%)	
LCC	117 (3.0%)	74 (2.1%)		56 (2.4%)	60 (2.6%)	
PSC	3 (0.1%)	2 (0.1%)		1 (0.0%)	0 (0.0%)	
Other	222 (5.8%)	210 (6.1%)		134 (5.7%)	136 (5.8%)	
Unknown	144 (3.7%)	115 (3.3%)		73 (3.1%)	79 (3.4%)	

*NSCLC* non-small cell lung cancer, *NLNs* the number of removed lymph nodes, *PSM* propensity score matching, Lung *NOS* uncertain location on lung, *Adeno* adenocarcinoma, *SCC* squamous cell carcinoma, *LCC* large cell carcinoma, *PSC* pulmonary sarcomatoid carcinoma, *Other* other histological types

^*^Chi-squared test

^**^Fisher’s exact test

^***^Student’s *t* test

### Effect of NLNs on survival outcomes

**Fig. 2:**
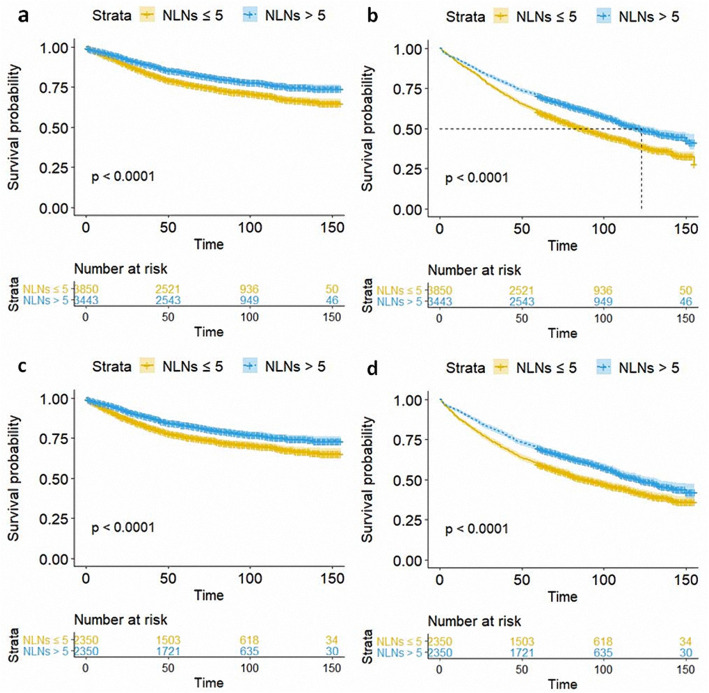
Kaplan–Meier curves showing the effect of NLNs on CSS (**a**) and OS (**b**) of unmatched patients with stage IA ≤ 2-cm NSCLC. Kaplan–Meier curves showing the effect of NLNs on CSS (**c**) and OS (**d**) of matched patients with stage IA ≤ 2-cm NSCLC

Kaplan–Meier analysis and log-rank tests were performed to investigate the effect of NLNs on CSS and OS in the eligible patients without and with PSM. The analysis revealed a common trend in the unmatched and matched cohorts; patients with > 5 NLNs (group B) showed significantly better CSS and OS than those patients with ≤ 5 NLNs (group A) (all *p* values < 0.001) (Fig. 2). Precisely, in the unmatched cohort, the 1-, 3-, and 5-year CSS of group A (NLNs ≤ 5) were 94.8%, 84.4%, and 77.3%, respectively, while the corresponding values of group B (NLNs > 5) were 96.6%, 89.5%, and 84.1%. Similarly, the 1-, 3-, and 5-year OS of group A (NLNs ≤ 5) were 90.2%, 73.4%, and 60.5%, respectively, while the corresponding values for group B (NLNs > 5) were 92.3%, 80.0%, and 70.4%. Likewise, after PSM, 1-, 3-, and 5-year CSS of group A (NLNs ≤ 5) were 93.3%, 83.0%, and 76.1%, respectively, while the corresponding values for group B (NLNs > 5) were 96.3%, 88.7%, and 83.3%. As for OS, 1-, 3-, and 5-year OS of group A (NLNs ≤ 5) were 87.4%, 71.5%, and 59.7%, respectively, while the corresponding values for group B (NLNs > 5) were 92.3%, 79.7%, and 69.7%.

To further validate the impact of NLNs on OS and CSS, univariable and multivariable analyses were conducted in the unmatched and matched cohorts. Without PSM, the univariable analysis revealed that NLNs, sex, age at diagnosis, TNM stage, grade, tumor size, surgical methods, and histological types were associated with CSS while NLNs, sex, age at diagnosis, TNM stage, grade, tumor location, tumor size, race/ethnicity, surgical methods, and histological types were associated with OS of the patients (Table 2). In addition, the multivariable analysis also revealed that NLNs, sex, age at diagnosis, grade, tumor size, surgical methods, and histological types independently predicted both CSS and OS (Table 2). After PSM, the univariable analysis confirmed that NLNs, sex, age at diagnosis, TNM stage, grade, tumor size, surgical methods, and histological types were statistically significant predictors for CSS and OS. In addition, the multivariable Cox regression analysis also revealed that NLNs, sex, age at diagnosis, grade, surgical methods, and histological types were independent predictors for CSS and OS (Table 3). In summary, univariable and multivariable analyses uniformly indicated that the NLNs were a significant and independent prognostic factor in the unmatched and matched patients.

**Table 2:** The association between NLNs and CSS together with OS in univariate and multivariate analyses without PSM

		CSS			OS		
Variable	Number	Univariate analysis	Multivariate analysis		Univariate analysis	Multivariate analysis	
		*p* value	HR (95%CIs)	*p* value	*p* value	HR (95%CIs)	*p* value
NLNs							
≤ 5	3850		1 (reference)			1 (reference)	
> 5	3443	< 0.001	0.791 (0.710–0.882)	< 0.001	< 0.001	0.821 (0.763–0.884)	< 0.001
Sex							
Male	3040		1 (reference)			1 (reference)	
Female	4253	< 0.001	0.757 (0.686–0.835)	< 0.001	< 0.001	0.723 (0.676–0.773)	< 0.001
Age							
≤ 65	2985		1 (reference)			1 (reference)	
> 65	4308	< 0.001	1.548 (1.393–1.720)	< 0.001	< 0.001	1.946 (1.806–2.096)	< 0.001
TNM stage							
IA1	1244		1 (Reference)			1 (Reference)	
IA2	6049	0.003	1.023 (0.843–1.243)	0.815	< 0.001	1.038 (0.911–1.182)	0.578
Grade							
I	1615		1 (reference)			1 (reference)	
II	3191	< 0.001	2.109 (1.794–2.479)	< 0.001	< 0.001	1.631 (1.471–1.807)	< 0.001
III	1839	< 0.001	2.810 (2.364–3.341)	< 0.001	< 0.001	2.004 (1.792–2.242)	< 0.001
IV	92	< 0.001	1.476 (0.911–2.392)	0.113	< 0.001	1.303 (0.944–1.797)	0.107
Unknown	556	< 0.001	1.785 (1.414–2.253)	< 0.001	< 0.001	1.407 (1.207–1.640)	< 0.001
Tumor location (%)							
Upper lobe	4547					1 (Reference)	
Middle lobe	459	0.597			0.017	0.895 (0.773–1.036)	0.138
Lower lobe	2181	0.794			0.712	1.038 (0.964–1.119)	0.320
Main bronchus	9	0.427			0.351	0.800 (0.257–2.485)	0.699
Overlapping	28	0.821			0.431	1.429 (0.859–2.378)	0.169
Lung NOS	69	0.176			0.148	1.114 (0.811–1.530)	0.506
Tumor size (mm)	–	< 0.001	1.022 (1.004–1.041)	0.015	< 0.001	1.018 (1.005–1.030)	0.005
Race							
American Indian	29					1 (Reference)	
Asian	397	0.725			0.160	0.613 (0.346–1.083)	0.092
Black	563	0.410			0.742	0.962 (0.551–1.679)	0.890
White	6291	0.548			0.747	0.920 (0.533–1.587)	0.764
Unknown	13	0.981			0.049	0.150 (0.020–1.150)	0.068
Surgical methods							
Wedge resection	1647		1 (reference)			1 (reference)	
Segmental resection	364	0.397	0.944 (0.757–1.178)	0.610	0.009	0.841 (0.721–0.981)	0.027
Lobectomy	5228	< 0.001	0.719 (0.637–0.812)	< 0.001	< 0.001	0.696 (0.641–0.756)	< 0.001
Pneumonectomy	45	0.033	1.957 (1.183–3.238)	0.009	0.030	1.623 (1.137–2.315)	0.008
Unknown	6	0.426	0.430 (0.060–3.061)	0.399	0.284	0.567 (0.182–1.763)	0.327
Histological types							
Adeno	4800		1 (reference)			1 (reference)	
SCC	1606	< 0.001	1.046 (0.926–1.182)	0.470	< 0.001	1.376 (1.271–1.490)	< 0.001
LCC	191	< 0.001	1.666 (1.276–2.175)	< 0.001	< 0.001	1.533 (1.257–1.869)	< 0.001
PSC	5	0.012	2.516 (0.808–7.832)	0.111	0.036	1.691 (0.633–4.517)	0.294
Other	432	0.011	1.241 (1.014–1.518)	0.036	0.020	1.140 (0.984–1.321)	0.080
Unknown	259	0.008	0.971 (0.755–1.247)	0.815	< 0.001	1.135 (0.961–1.339)	0.136

*NSCLC* non-small cell lung cancer, *NLNs* the number of removed lymph nodes, *PSM* propensity score matching, *HR* hazard ratio, *CIs* confident intervals, Lung *NOS* uncertain location on lung, Adeno adenocarcinoma, *SCC* squamous cell carcinoma, *LCC* large cell carcinoma, *PSC* pulmonary sarcomatoid carcinoma, *Other* other histological types

**Table 3:** The association between NLNs and CSS together with OS in univariate and multivariate analyses with PSM

		CSS			OS		
Variable	Number	Univariate analysis	Multivariate analysis		Univariate analysis	Multivariate analysis	
		*p* value	HR (95%CIs)	*p* value	*p* value	HR (95%CIs)	*p* value
NLNs							
≤ 5	2350		1 (reference)			1 (reference)	
> 5	2350	< 0.001	0.631 (0.557–0.714)	< 0.001	< 0.001	0.650 (0.597–0.708)	< 0.001
Sex							
Male	1908		1 (reference)			1 (reference)	
Female	2790	< 0.001	0.631 (0.557–0.714)	< 0.001	< 0.001	0.714 (0.657–0.777)	< 0.001
Age							
≤ 65	1975		1 (reference)			1 (reference)	
> 65	2725	< 0.001	1.410 (1.244–1.599)	< 0.001	< 0.001	1.829 (1.671–2.002)	< 0.001
TNM stage							
IA1	699		1 (reference)			1 (reference)	
IA2	4001	0.005	1.153 (0.900–1.478)	0.261	0.003	1.117 (0.944–1.322)	0.197
Grade							
I	1055		1 (reference)			1 (reference)	
II	2044	< 0.001	2.094 (1.724–2.543)	< 0.001	< 0.001	1.642 (1.446–1.864)	< 0.001
III	1187	< 0.001	2.762 (2.242–3.402)	< 0.001	< 0.001	2.055 (1.791–2.360)	< 0.001
IV	64	0.007	1.574 (0.901–2.748)	0.111	0.007	1.248 (0.843–1.847)	0.268
Unknown	350	0.005	1.643 (1.222–2.210)	0.001	0.037	1.277 (1.045–1.560)	0.017
Tumor location (%)							
Upper lobe	2783						
Middle lobe	294	0.640			0.158		
Lower lobe	1560	0.627			0.443		
Main bronchus	2	0.982			0.973		
Overlapping	20	0.891			0.977		
Lung NOS	41	0.079			0.230		
Tumor size (mm)	–	< 0.001	1.019 (0.997–1.042)	0.094	< 0.001	1.014 (0.999–1.030)	0.066
Race							
American Indian	22						
Asian	289	0.820			0.225		
Black	393	0.455			0.865		
White	3989	0.667			0.924		
Unknown	7	0.978			0.967		
Surgical methods							
Wedge resection	460		1 (reference)			1 (reference)	
Segmental resection	158	*0.025*	0.546 (0.392–0.761)	< 0.001	< 0.001	0.503 (0.396–0.638)	< 0.001
Lobectomy	4060	< 0.001	0.339 (0.283–0.405)	< 0.001	< 0.001	0.353 (0.312–0.400)	< 0.001
Pneumonectomy	22	0.533	0.581 (0.256–1.318)	0.194	0.225	0.537 (0.301–0.958)	0.035
Unknown	0	NA	NA	NA			
Histological types							
Adeno	3157		1 (reference)			1 (reference)	
SCC	1004	< 0.001	1.064 (0.915–1.237)	0.424	< 0.001	1.371 (1.240–1.516)	< 0.001
LCC	116	< 0.001	1.640 (1.163–2.314	0.005	< 0.001	1.585 (1.228–2.045)	< 0.001
PSC	1	0.981	0.000 (0.000–Inf)	0.981	0.981	0.000 (0.000–Inf)	0.972
Other	270	0.140	1.140 (0.883–1.472)	0.314	0.082	1.117 (0.929–1.342)	0.240
Unknown	152	0.575	0.867 (0.612–1.228)	0.422	0.008	1.086 (0.867–1.361)	0.472

*NSCLC* non-small cell lung cancer, *NLNs* the number of removed lymph nodes, *PSM* propensity score matching, *HR* hazard ratio, *CIs* confident intervals, *Lung NOS* uncertain location on lung, *Adeno* adenocarcinoma, *SCC* squamous cell carcinoma, *LCC* large cell carcinoma, *PSC* pulmonary sarcomatoid carcinoma, *Other* other histological types, *Inf* infinity

**Fig. 3:**
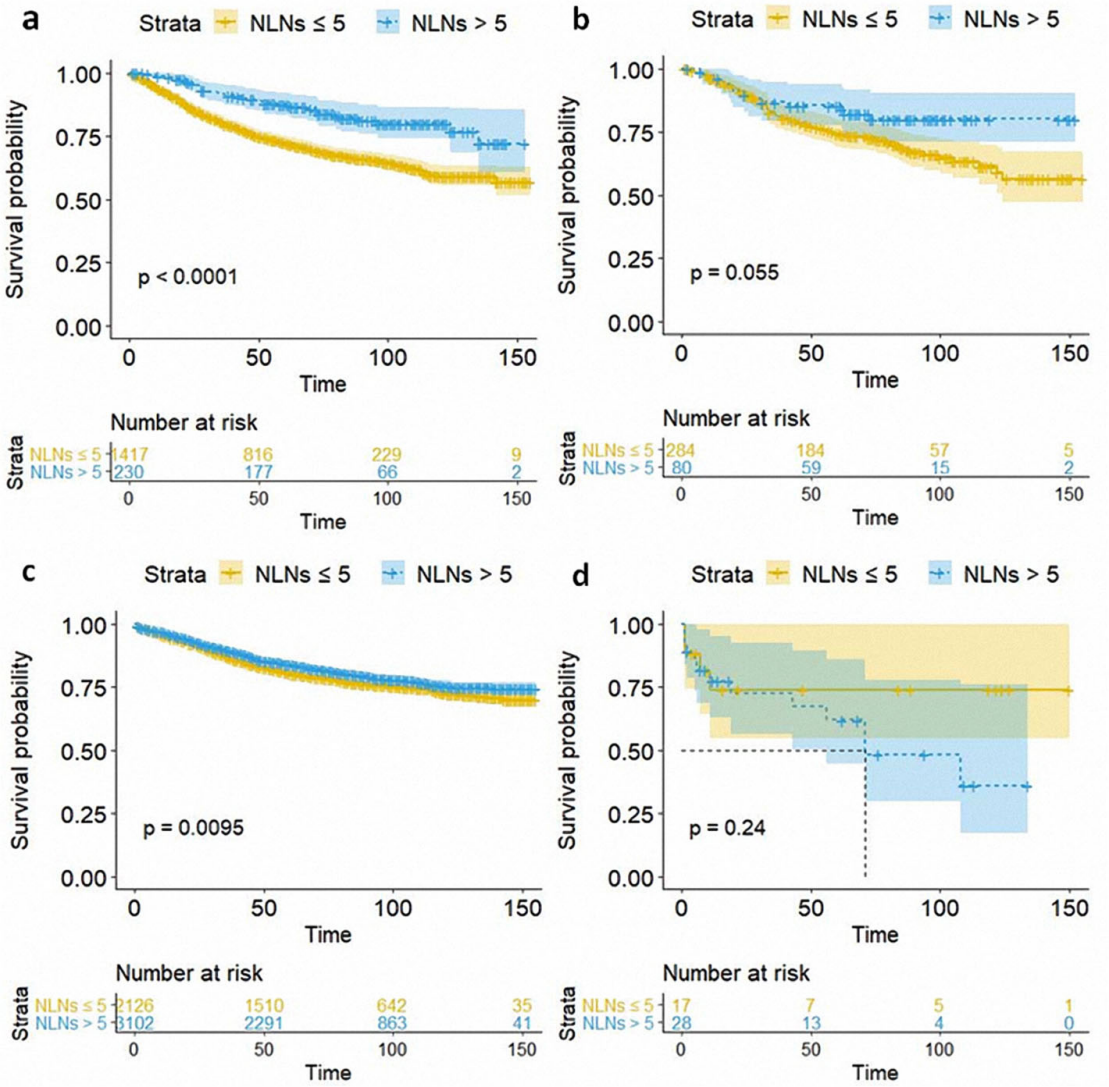
Kaplan–Meier curves showing CSS for patients with NLNs ≤ 5 and > 5 who underwent wedge resection (**a**), segmental resection (**b**), lobectomy (**c**), and pneumonectomy (**d**)

**Fig. 4:**
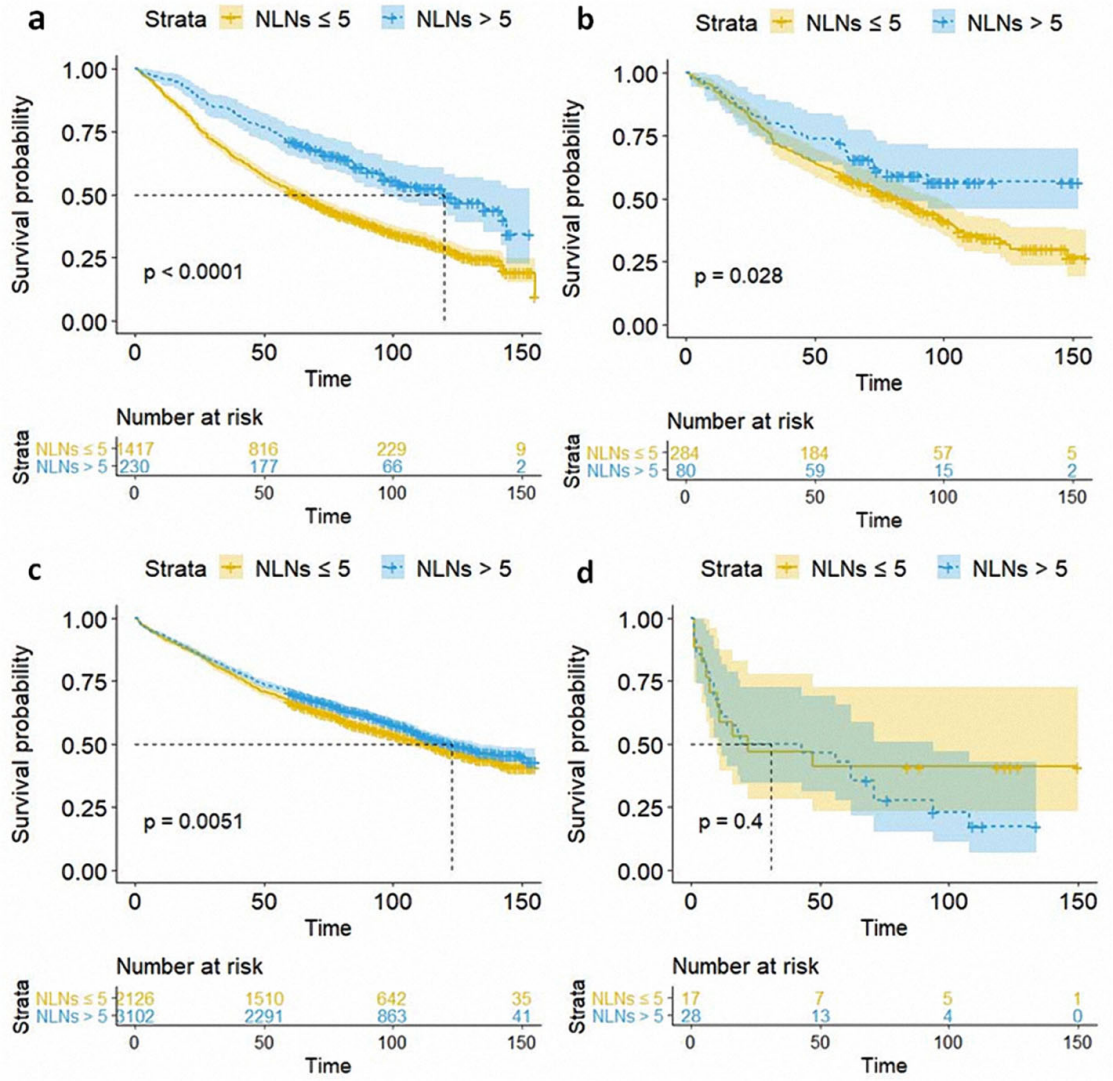
Kaplan–Meier curves showing OS for patients with NLNs ≤ 5 and > 5 who underwent wedge resection (**a**), segmental resection (**b**), lobectomy (**c**), and pneumonectomy (**d**)

Besides, the patients who underwent lobectomy or wedge resection and with more than 5 LNs resected had a significantly better CSS (*P* < 0.001, *P* = 0.0095, respectively), while no significant impact on conferring better CSS was observed for segmental resection, and pneumonectomy, although the *p* values of segmental resection were near 0.05 (Fig. 3). Meanwhile, the number of dissected LNs contributed to the better OS of patients who underwent wedge resection, segmental resection, and lobectomy, (*P* < 0.001, *P* = 0.028, *P* = 0.0051, respectively) (Fig. 4).

### Subgroup analysis of survival outcomes

**Fig. 5:**
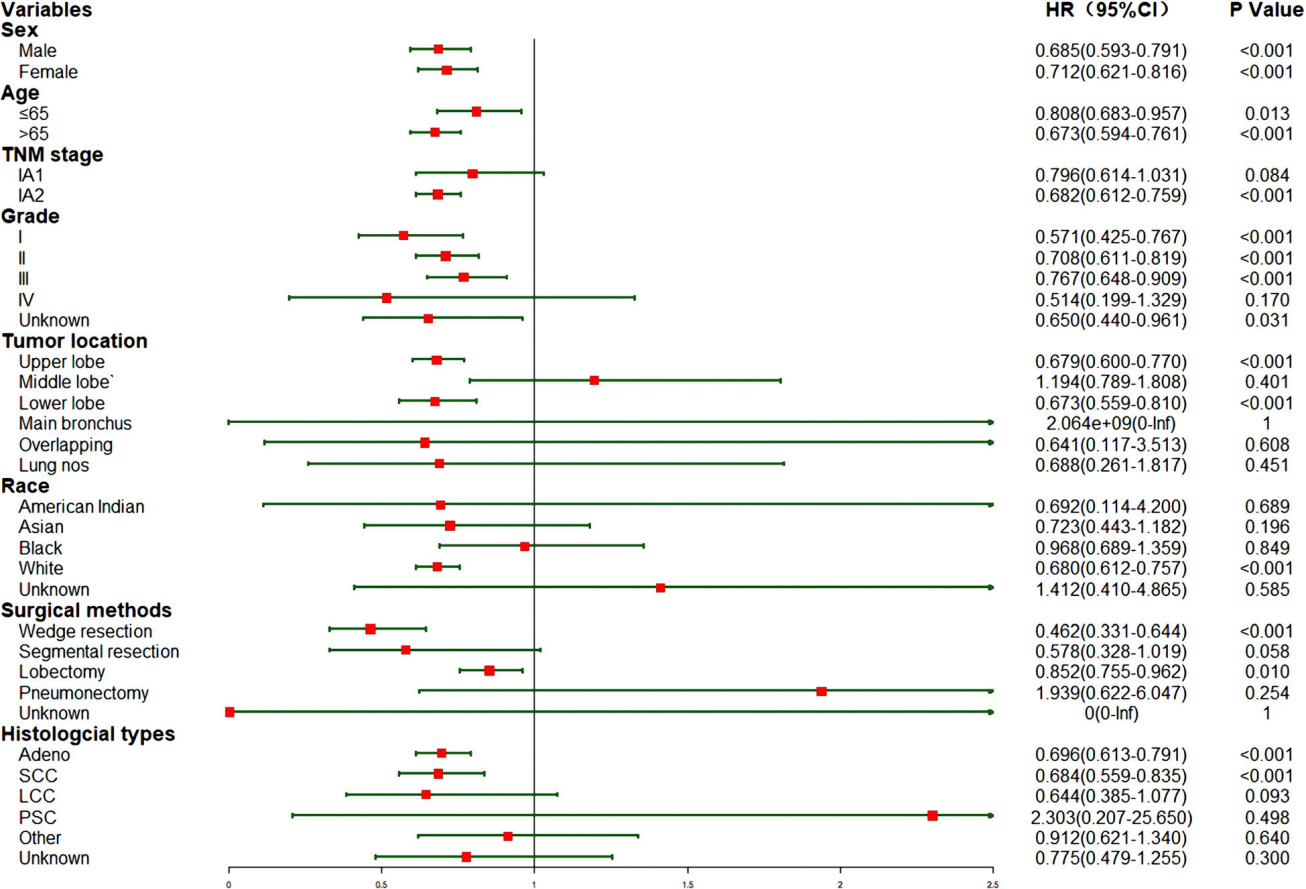
The subgroup analysis of this unmatched cohort investigating the effect on CSS

**Fig. 6:**
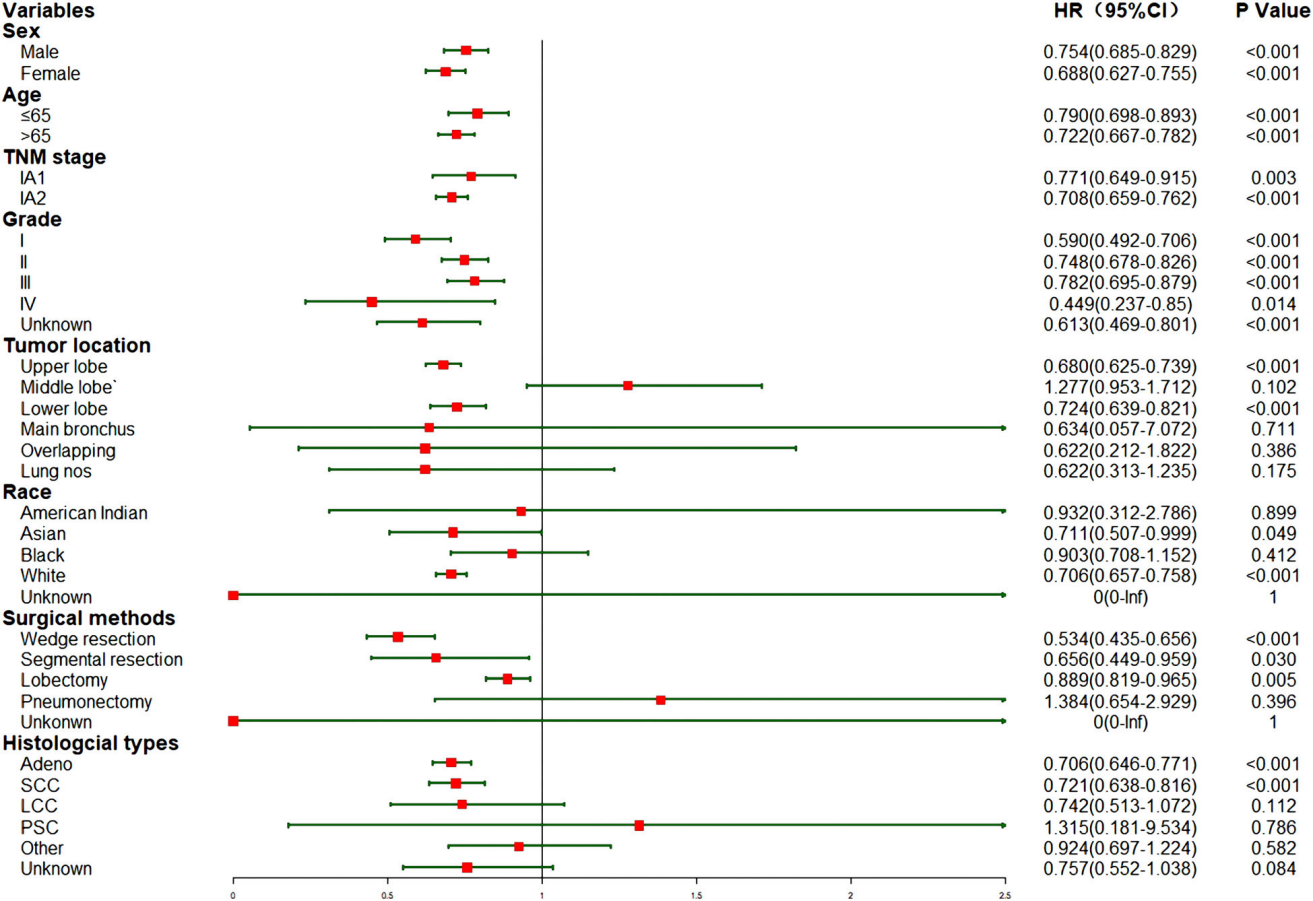
The subgroup analysis of this matched cohort investigating the effect on OS

Furthermore, a subgroup analysis was conducted to investigate the effect of listed factors in Table 1 on CSS and OS of eligible patients (without PSM). The effects of the rest of the variables on CSS and OS were illustrated in Figs. 5 and 6, respectively.

## Discussion

This study examined the relationship between NLNs and the survival outcomes of patients with stage IA NSCLC ≤ 2 cm in size who underwent different types of lung surgeries. Kaplan–Meier analysis and log-rank test demonstrated that patients with more than 5 LNs dissected showed significantly better CSS and OS in the unmatched and matched patients, which indicates that dissection of more LNs might result in better survival. In addition, both univariable and multivariable analyses revealed that NLNs served as a protective prognostic predictor for CSS and OS in the unmatched and matched patients. Further, according to our subgroup study, patients who underwent wedge resection, segmental resection, or lobectomy rather than pneumonectomy, together with resection of more than 5 LNs, had statistically better survival outcomes. To sum up, patients with stage IA NSCLC ≤ 2 cm in size who undergo wedge resection, segmental resection, or lobectomy may have better survival outcomes after undergoing incremental NLNs.

To date, the relationship between NLNs and survival outcome for patients with stage IA NSCLC ≤ 2 cm in size remains controversial. Dissection of more LNs may result in a clearer TNM classification, a higher possibility to discover, and eradicate occult metastasis, and therefore a better survival outcome [9, 10]. Further, Burdett et al. noted that an increase in NLNs can make the pTNM stage more precise that it is beneficial to decide the strategy of adjuvant therapy [15]. Several retrospective studies based on the SEER database have confirmed the relationship between NLNs and survival outcomes of patients with stage N0 NSCLC and have proposed optimal NLNs for survival outcomes [4, 6–8]. Furthermore, Osarogiagbon et al. suggested that the resection of 11 to 15 LNs might be associated with better survival [6]. Similarly, Becker et al. observed a consistently increasing survival benefit for patients with 16 to 18 LNs removed [8]. Additionally, several single-center studies also highlighted the relationship. However, a study conducted by Saji et al. determined the optimal minimum NLNs as 8 for patients with stage I NSCLC [16]. Moreover, Wen et al. demonstrated that patients with stage T2N0M0 NSCLC should have at least 12 LNs resected during the surgery [5]. In addition, the minimal NLNs for patients with N0 NSCLC was not clear. Likewise, the NCCN guidelines indicate that N1 and N2 nodes should be dissected, and mapping should be performed; however, it does not specify the minimum NLNs [17]. Conversely, the European Society of Thoracic Surgeons guidelines proposes that at least six LNs from the hilar and mediastinal stations should be removed for accurate nodal staging [10].

Unfortunately, only a few studies have focused on whether NLNs is associated with the survival outcomes of patients with stage IA NSCLC 2 cm or less. Ding et al. demonstrated that 4 to 16 LNs should be examined for patients with NSCLC < 2 cm undergoing wedge resection, and these NLNs could provide a survival advantage to those patients. However, among patients who received segmentectomy, LN resection did not improve survival outcomes [7]. In Ding’s study, there were some stage IB patients, as they did not exclude the patients with pleura invasion. Wolf et al. showed that lobectomy led to superior survival compared to sub-lobar resection in patients with N0 NSCLC ≤ 2 cm; however, the survival outcome was not different from that in patients with LN resection performed together with lobectomy [18]. In contrast, our study revealed that LN resection conferred independent survival benefit to patients with NSCLC ≤ 2 cm, and NLNs contributed to survival benefit for patients who received wedge resection, segmental resection, or lobectomy, but not for patients who received pneumonotomy. Several theories can explain this phenomenon. There were only 45 patients who underwent pneumonectomy in comparison to a larger number of patients who received wedge resection, segmental resection, or lobectomy in our study (*n* = 1647, 364, 5228, respectively), and a small sample entails a risk of bias. Notably, the patients who received lobectomy or wedge resection and with more than 5 LNs dissected had a significantly better CSS, while there was no effect of dissection of more than 5 LNs with segmental resection, or pneumonectomy on CSS. The smaller sample sizes of patients undergoing segmental resection, or pneumonectomy might be responsible for it. Since p values of segmental resection were both near 0.05 (*P* = 0.055), and univariable and multivariable studies together with a subgroup study on OS revealed that all the four surgical methods except pneumonectomy together with dissection of more than 5 LNs contributed to better survival outcome, we concluded that patients who underwent wedge resection, segmental resection, or lobectomy had better survival outcome after having incremental NLNs.

There are several limitations to this study. Because of the retrospective nature of the study, the bias in our results was inevitably unremovable. However, the potential selection bias derived from a retrospective study was minimized by performing PSM. Due to the short follow-up period and a low malignancy rate of stage IA NSCLC tumors ≤ 2 cm, the median survival time of all patients was not identified. The exact location of the dissected LNs was unknown due to the statistical limitations. Moreover, our findings did not provide an exact optimal NLNs for LN dissection in clinical practice. Despite these limitations, we believe that our study could be used as a reference for the treatment of patients with stage IA NSCLC ≤ 2 cm in size who undergo wedge resection, segmental resection, or lobectomy.

## Conclusions

In conclusion, NLNs was a strong prognosticator for patients who received wedge resection, segmental resection, or lobectomy rather than pneumonectomy. Our study indicates that patients with stage IA NSCLC ≤ 2 cm who undergo wedge resection, segmental resection, or lobectomy should undergo higher NLNs to achieve better OS, but the efficacy of our conclusions should be investigated further by a large-scale, prospective, multi-center study.

## Data Availability

Please contact author for data requests.

## Abbreviations

NSCLC: Non-small cell lung cancer
NLNs: The number of removed lymph nodes
CSS: Cancer-specific survival
OS: Overall survival
SEER: Surveillance, Epidemiology, and End Results database
PSM: Propensity score matching
AJCC: American Joint Committee on Cancer
LNs: Lymph nodes
CT: Computed tomography
LC: Lung cancer
HR: Hazard ratio
CIs: Confidence intervals
SD: Standard deviation
Lung NOS: Uncertain location on lung
Adeno: Adenocarcinoma
SCC: Squamous cell carcinoma
LCC: Large cell carcinoma
PSC: Pulmonary sarcomatoid carcinoma
Other: Other histological types
Inf: Infinity
